# Improving estimation of Parkinson’s disease risk—the enhanced PREDICT-PD algorithm

**DOI:** 10.1038/s41531-021-00176-9

**Published:** 2021-04-01

**Authors:** Jonathan P. Bestwick, Stephen D. Auger, Cristina Simonet, Richard N. Rees, Daniel Rack, Mark Jitlal, Gavin Giovannoni, Andrew J. Lees, Jack Cuzick, Anette E. Schrag, Alastair J. Noyce

**Affiliations:** 1 Preventive Neurology Unit, Wolfson Institute of Preventive Medicine, Barts and the London School of Medicine and Dentistry, Queen Mary University of London, London, UK; 2 Department of Clinical and Movement Neuroscience, UCL Institute of Neurology, University College London, London, UK; 3 Barts and The London School of Medicine and Dentistry, Queen Mary University, London, UK; 4 Blizard Institute, Barts and the London School of Medicine and Dentistry, Queen Mary University, London, UK

**Keywords:** Predictive markers, Risk factors

## Abstract

We previously reported a basic algorithm to identify the risk of Parkinson’s disease (PD) using published data on risk factors and prodromal features. Using this algorithm, the PREDICT-PD study identified individuals at increased risk of PD and used tapping speed, hyposmia and REM sleep behaviour disorder (RBD) as “intermediate” markers of prodromal PD in the absence of sufficient incident cases. We have now developed and tested an enhanced algorithm which incorporates the intermediate markers into the risk model. Risk estimates were compared using the enhanced and the basic algorithm in members of the PREDICT-PD pilot cohort. The enhanced PREDICT-PD algorithm yielded a much greater range of risk estimates than the basic algorithm (93–609-fold difference between the 10th and 90th centiles vs 10–13-fold respectively). There was a greater increase in the risk of PD with increasing risk scores for the enhanced algorithm than for the basic algorithm (hazard ratios per one standard deviation increase in log risk of 2.75 [95% CI 1.68–4.50; *p* < 0.001] versus 1.47 [95% CI 0.86–2.51; *p* = 0.16] respectively). Estimates from the enhanced algorithm also correlated more closely with subclinical striatal DaT-SPECT dopamine depletion (*R*^2^ = 0.164, *p* = 0.005 vs *R*^2^ = 0.043, *p* = 0.17). Incorporating the previous intermediate markers of prodromal PD and using likelihood ratios improved the accuracy of the PREDICT-PD prediction algorithm.

## Introduction

Neurodegeneration preceding a formal diagnosis of Parkinson’s disease (PD) is associated with identifiable motor and non-motor features. Evidence-based algorithms have been developed to try to identify individuals in this pre-diagnostic phase according to exposure to common risk factors, the presence of early clinical features and using simple screening tests. Two notable approaches to risk estimation are the PREDICT-PD algorithm^1^ and the MDS prodromal PD research criteria^2,3^.

The PREDICT-PD study initially ran as a prospective pilot study in 60–80-year olds^4^, and was designed to estimate risk from information gathered using simple online tests and remotely administered screening tools, including demographic information, environmental exposures and early symptoms identified in a systematic review and meta-analysis^5^. Reduced finger tapping speed on a keyboard tapping task, hyposmia and probable REM sleep behaviour disorder (RBD) were used as “intermediate” markers or outcomes to indicate the possibility of prodromal PD^1^. The MDS research criteria for prodromal PD, first published in 2015, incorporate additional clinical and radiological tests, as well as these three markers. As participants of the ongoing longitudinal PREDICT-PD pilot cohort develop PD, it is possible to use incident diagnosis of PD as the outcome and improve the algorithm by incorporating intermediate markers into risk estimates.

PREDICT-PD risk estimation has previously been based upon odds ratios. This had the limitation that if a risk factor is known to be absent, there is no adjustment to the change in risk that this represents. Whilst it is expected that the ranking of risk estimates would be the same through either method, using likelihood ratios (LRs) instead of odds ratios allows better characterisation of overall risk, as the presence (LR+) or absence (LR−) of each individual marker modifies risk estimates in the algorithm.

Here, we sought to refine the PREDICT-PD algorithm, first by changing the method of risk estimation from odds ratios to likelihood ratios and then by incorporating objective assessment of tapping speed and smell, and probable RBD into risk estimates. To assess whether these steps improved risk estimation, we compared the distributions of risk derived from the enhanced algorithm to those using the basic PREDICT-PD algorithm^1,4^, and also to the MDS prodromal criteria algorithm^2,3^. We then considered the members of the PREDICT-PD pilot cohort who have developed PD to date and assessed risk estimation preceding formal diagnosis. Finally, we assessed the relationship between risk estimates and subclinical striatal dopamine depletion measured by dopamine transporter imaging (DaT-SPECT) in a subgroup of the participants who have previously been reported using the basic algorithm^6^.

## Results

### The enhanced PREDICT-PD algorithm

New likelihood ratios for risk factors which had not previously been calculated are presented in Table 1, together with the prevalence and odds ratio data used to make these calculations. All the factors which were available for inclusion into risk estimates in either the basic or enhanced PREDICT-PD risk estimates are outlined in Table 2, alongside the most recent MDS prodromal criteria likelihood ratios^3^. No prevalence data were available for pesticide exposure or having a 1st degree relative with PD, so negative likelihood ratios for these factors could not be calculated.

**Table 1:** Prevalence, odds ratios^5^ and likelihood ratios for a positive (LR + ) or negative (LR-) association with PD for risk factors (ever versus never) without previous estimates.

Risk factor	Prevalence	Odds Ratio	LR+	LR−
Head injury	0.03	1.58	1.55	0.98
NSAID use	0.80	0.83	0.96	1.16
CCB use	0.43	0.90	0.94	1.04
Beta blocker use	0.28	1.28	1.19	0.93
Alcohol use	0.80	0.90	0.98	1.09

**Table 2:** Likelihood ratios (LRs) and odds ratios (ORs) for PD risk factors collected in the PREDICT-PD pilot cohort using the PREDICT-PD risk algorithms and MDS prodromal criteria.

Factor	MDS (LRs)^a^	Basic PREDICT (ORs)	Enhanced PREDICT (LRs)
*Factors in both MDS and PREDICT algorithms*			
Age	Categorical 5-year age intervals	Age-based equation	Age-based equation
Sex	Male 1.2, female 0.8	Female 0.67	Male 1.2, Female 0.8
Coffee use	LR+=0.88, LR−=1.35	0.67	LR+=0.88, LR−=1.35
Current smoker	LR+=0.51	0.44	LR+=0.51
Former smoker	LR+=0.91	0.78	LR+=0.91
1st degree relative	LR+=2.5	3.2	LR+=2.5
Constipation	LR+=2.5, LR−=0.82	2.3	LR+=2.5, LR−=0.82
Erectile Dysfunction	LR+=3.4, LR−=0.87	3.8	LR+=3.4, LR−=0.87
Depression/anxiety	LR+=1.6, LR−=0.88	1.86	LR+=1.6, LR−=0.87
*Factors in one algorithm but not the other*			
Objective motor impairment^b^	LR+=3.5, LR−=0.60	–	Bivariate Gaussian model based equation^c^
REM-sleep behaviour disorder^b^	LR+=2.8, LR−=0.89	–	LR+=2.8, LR−=0.89
Olfactory impairment^b^	LR+=4.0, LR−=0.43	–	Logistic regression model based equation^c^
Pesticides exposure	LR+=1.5	–	LR+=1.5
Never smoked	LR+=1.2	–	LR+=1.25
Diabetes	LR+=1.50, LR−=0.97		LR+=1.50, LR−=0.97
Head injury	–	1.58	LR+=1.55, LR−=0.98
NSAID use	–	0.83	LR+=0.96,LR−=1.16
CCB use	–	0.9	LR+=0.94, LR−=1.04
Beta blocker use	–	1.28	LR+=1.19, LR−=0.93
Alcohol	–	0.9	LR+=0.98, LR−=1.09

^a^Berg et al. ^2^, Heinzel et al. ^3^

^b^Previously used as intermediate outcome markers and so did not feature in the basic PREDICT-PD algorithm.

^c^Bestwick et al. ^8^

Supplementary Fig. 1 shows the risk (expressed as an odds) according to age for the basic PREDICT-PD algorithm and the revised risk according to age for the enhanced PREDICT-PD algorithm (based on the risk according to age categories in the MDS criteria)^2,3^. The revised equation for estimating age-related risk is given by Eq. (1).

1 $${\rm{Odds}} = 1:22.098 + 78.900{\rm{e}}^{ - 0.14053\left( {{\rm{age}} - 60} \right)}$$

Between the ages of 60 and 70 years, the old and revised equations estimated near identical risk. There were some minor differences at higher ages however. For example, the revised equation gave a risk for an 80-year old of 1:27, which is higher than the 1:31 risk for the previous equation.

### Distributions of risk

**Fig. 1:**
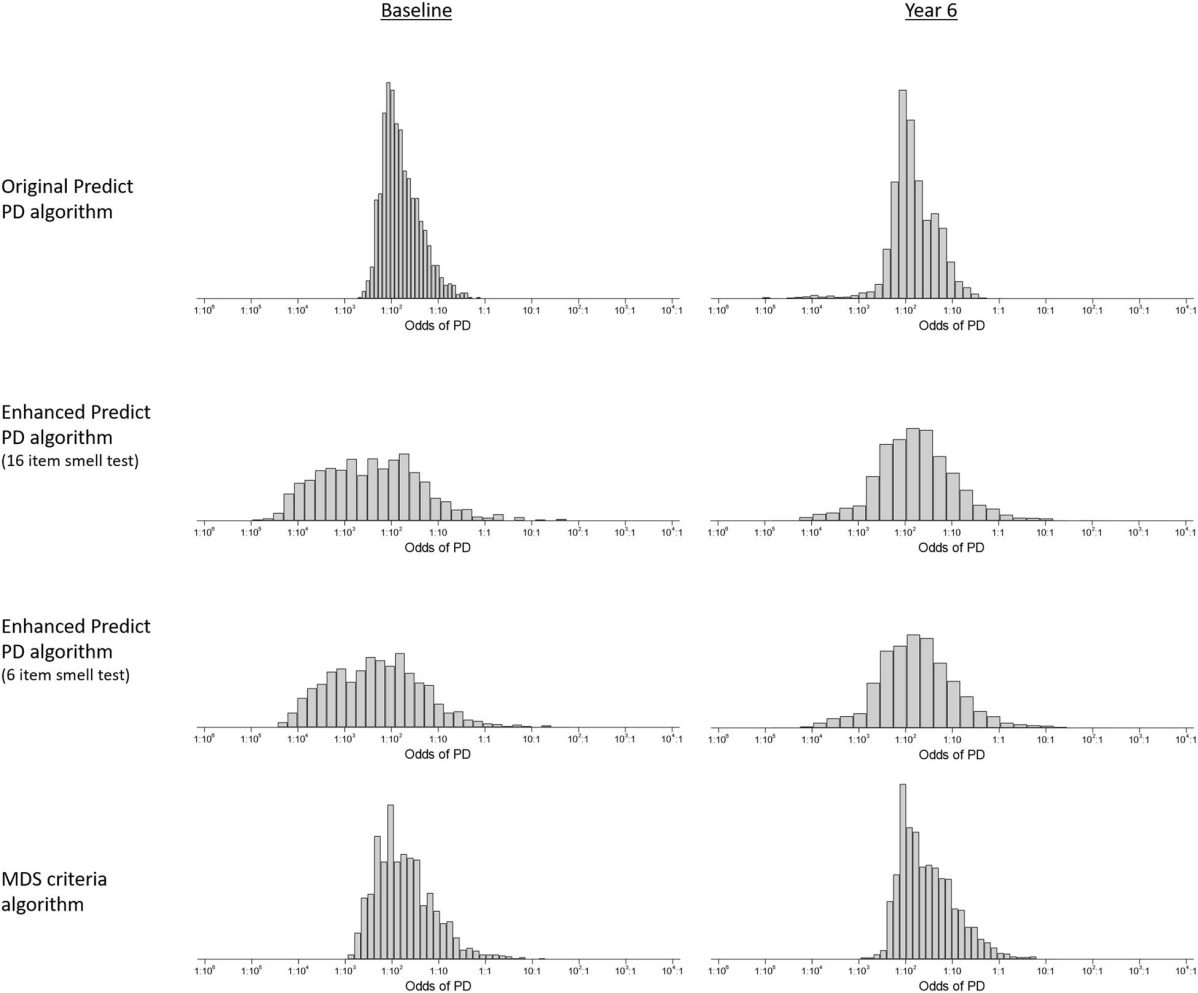
Histograms of risk scores for PREDICT-PD participants (presented as odds). Left column shows baseline risks and the right column show risks for the latest survey year. Risks calculated using the basic PREDICT-PD algorithm, the enhanced PREDICT-PD algorithm using a 16 item smell test, the enhanced PREDICT-PD algorithm using a 6-item smell test and the MDS criteria algorithm are shown on the first, second, third and fourth rows respectively.

Figure 1 shows histograms of risk (expressed as odds) at baseline and year 6 of follow-up. Supplementary Figure 2 shows histograms for all years. Shown in the figures are the distributions using the basic PREDICT-PD algorithm, the enhanced PREDICT-PD algorithm (using either the most discriminant 16 or 6-items from the 40-item UPSIT) and the MDS prodromal criteria algorithm. Supplementary Table 2 shows selected centiles of risk for each algorithm and survey year. Figure 1 (and Supplementary Fig. 2) shows that for each survey year, the basic PREDICT-PD algorithm yields the least spread in risk, with the enhanced PREDICT-PD algorithm producing greater spread (similar spread whether the 16-item or the 6-item smell test subset were used for risk estimation). Over 6 time points, there was between a 10 and 13-fold difference in risk from the 10th to 90th centile of risk for the basic PREDICT-PD algorithm, compared to between a 93 and 609-fold difference in risk for the enhanced PREDICT-PD algorithm using a 16-item smell test, between a 90 and 487-fold difference in risk for the enhanced PREDICT-PD algorithm using a 6-item smell test, and between a 33 and 42-fold difference in risk for the MDS criteria algorithm. Between the 25th and 75th centile of risk, the respective fold differences were between 3.5 and 4.3, between 11 and 41, between 11 and 28, and between 6.4 and 7.2.

### Comparison of PREDICT-PD risk scores in participants diagnosed with PD

Ten people in the PREDICT-PD pilot cohort have been diagnosed with PD to date. Supplementary Table 3 shows baseline demographic data and baseline risk scores for the ten. Table 3 shows the relationship between baseline risk score and incident PD using a Cox proportional hazards model. For both a 10-fold increase in risk and a one standard deviation increase in log risk, the hazard ratios for each risk algorithm were broadly similar; but whilst both the enhanced PREDICT-PD (using the 16-item smell test or the 6-item smell test) and the MDS prodromal criteria algorithms had strong associations with an increased risk of PD (*p* < 0.001, *p* < 0.001 and *p* = 0.001 respectively), the evidence of an association for the basic PREDICT-PD score was weak (*p* = 0.157). The enhanced PREDICT-PD algorithms and MDS prodromal criteria algorithm gave similar results, but it should be noted that only four of the ten incident PD cases had UPSIT scores at baseline, so for the remaining six, UPSIT scores were not included in the risk estimates. Furthermore, one of the six incident PD cases who did not have an UPSIT score at baseline, also did not complete the BRAIN test at baseline so could not have objective motor impairment included in the calculation of risk estimates.

**Table 3:** Hazard ratios (HR) of incident PD at 6 years of follow-up for a 10-fold increase in baseline risk and a one standard deviation (SD) increase in baseline log risk according to risk algorithm.

Algorithm	HR per 10-fold increase in risk (95% CI)	HR per SD of log risk (95% CI)	*p*-value
Basic PREDICT-PD	2.58 (0.69–9.56)	1.47 (0.86–2.51)	0.157
Enhanced PREDICT-PD (16-item smell test)	2.55 (1.62–4.01)	2.75 (1.68–4.50)	<0.001
Enhanced PREDICT-PD (6-item smell test)	2.62 (1.63–4.21)	2.62 (1.63–4.23)	<0.001
MDS prodromal criteria	3.11 (1.53–6.30)	2.04 (1.10–3.18)	0.002

### Comparison of risk estimates with DaT-SPECT binding

**Fig. 2:**
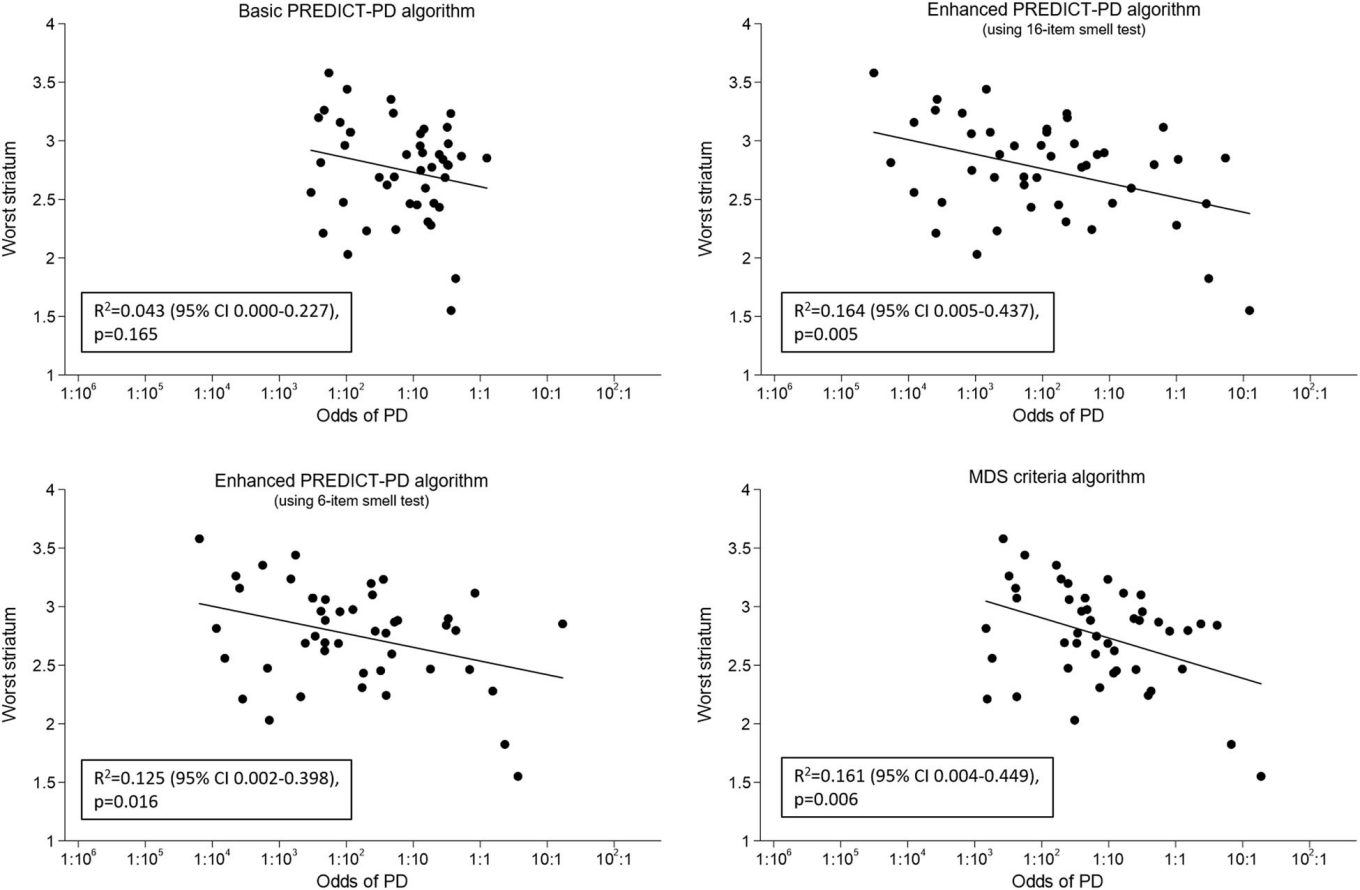
Scatterplots and regression lines of risk estimates (presented as odds) against striatal binding ratios on DaT-SPECT imaging. Risks calculated using the basic PREDICT-PD algorithm, the enhanced PREDICT-PD algorithm using a 16 item smell test, the enhanced PREDICT-PD algorithm using a 6-item smell test and the MDS prodromal criteria algorithm are shown in the top left, top right, bottom left and bottom right panels of the figure respectively.

Figure 2 shows the linear relationship between risk using each algorithm and striatal dopamine binding ratios with DaT-SPECT imaging in a subgroup of 46 individuals from the PREDICT-PD pilot cohort, none of whom had been diagnosed with PD. The figure shows that for each algorithm striatal binding values were lower as risks became higher; this relationship was not statistically significant for the basic PREDICT-PD algorithm (*R*^2^ = 0.043 [95% CI 0.000–0.227], *p* = 0.165), but did reach statistical significance for the enhanced PREDICT-PD algorithm using the 16-item smell test (*R*^2^ = 0.164 [95% CI 0.005–0.437], *p* = 0.005), the enhanced PREDICT-PD algorithm using the 6-item subset (*R*^2^ = 0.125 [95% CI 0.002–0.398], *p* = 0.016) and for the MDS prodromal criteria algorithm (*R*^2^ = 0.161 [95% CI 0.004–0.449], *p* = 0.006). The risk estimates from the enhanced PREDICT-PD and MDS criteria algorithms bear closer relation to striatal dopaminergic depletion, even in individuals who do not have a clinical PD diagnosis, compared with the basic PREDICT-PD algorithm; however, the *R*^2^ values were low in general. There was no evidence of a non-linear relationship for any of the algorithms.

## Discussion

Despite just ten people in the PREDICT-PD pilot cohort receiving a formal diagnosis of PD, the difference in relationship between baseline risk and incident PD between the basic and enhanced PREDICT-PD algorithms suggests a meaningful improvement in risk estimation. There was also a much greater range of risk estimates using the enhanced PREDICT-PD algorithm. Further evidence of the enhanced algorithm’s ability to identify those at risk of subsequent PD comes from the analysis of DaT-SPECT data, where the enhanced algorithm’s risk estimates bore a significant and closer relation to striatal dopamine depletion even when clinical PD was not evident. As the PREDICT-PD cohort now expands to a target of 10,000 participants and more data accrues for incident cases, for longer durations prior to PD diagnosis, the true temporal performance of enhanced risk prediction will become clearer.

The main alternative to the PREDICT-PD approach to risk estimation is the MDS research criteria for prodromal PD^2,3^. There is a suggestion from the data presented here that the enhanced PREDICT-PD algorithm has the potential to perform better than the MDS prodromal criteria algorithm since a greater range of risk estimates are likely to lead to greater accuracy and greater discrimination between those who do and do not develop PD, although the number of PD cases in the PREDICT-PD pilot cohort is so far too small to formally test this.

The main differences between how the two algorithms derive risk estimates are in the range of risk factors included and how risk according to age, smell and motor impairment are defined. The MDS prodromal criteria determine age-related risk according to which 5-year age interval a person falls into, whereas the enhanced PREDICT-PD algorithm uses a continuous function to assign risk according to exact age, because categorising a continuous variable leads to a loss of information^7^. Similarly, for smell and motor impairment, the MDS criteria use the binary presence or absence of a risk factor, rather than identifying specific items in the UPSIT that are associated with PD and generating likelihood ratios accordingly, or by treating motor impairment as a continuous variable as was done for the enhanced PREDICT-PD algorithm. The MDS criteria do include a wider range of radiological and clinical measures which provide important information, but require more intensive assessment than the simple, remotely administered tests used in PREDICT-PD. The MDS prodromal criteria do not consider head injury or the use of NSAIDs, calcium channel blockers, beta blockers or alcohol. We propose these as potentially valuable additions. A history of head injury might be particularly relevant; a systematic review found the odds ratio for head injury in PD to be 1.58^5^. Another difference between the MDS and PREDICT-PD approaches is their respective reporting of total combined risk in terms of percentage probability and odds (not to be confused with odds *ratios* for individual factors). It is possible to convert between the two, but here we favour expressing risk in terms of odds due to the resultant lower skew when plotting distributions on a log scale. Odds also has the advantage of allowing larger fold-changes between the highest and lowest risks to be demonstrated; their range is infinite rather than the bounding of percentage probabilities between 0 and 100. If such algorithms were to be used to report the risk of developing PD to individuals in the future, studies would be needed to determine preference in terms of how risk is reported

In the enhanced PREDICT-PD algorithm, we used the 16 smells that are present in both the US and UK UPSIT and are predictive of PD case status, and a subset of the 6 odours most strongly associated with PD^8^. While the enhanced PREDICT-PD algorithm with the 16 items led to the greatest range of risks, the enhanced PREDICT-PD algorithm with the 6 item test led to greater range of risks than the basic PREDICT-PD algorithm and the MDS prodromal criteria algorithm. The potential cost savings of a 6 item test may outweigh the benefits of an increased range of risks arising from use of either the full 40-item UPSIT or a 16 item test.

A limitation of both approaches is that the models currently assume a level of independence of risk factors which is unlikely to be true in reality. Models such as PREDICT-PD or the MDS prodromal criteria act as a first approximation to risk estimation but will need to be refined once prospective observational cohorts, such as the PREDICT-PD, the PARS^9^ and the Bruneck^10^ studies mature and provide more information regarding correlations between risk factors in the years preceding formal diagnosis with PD. This might allow risk estimation algorithms to evolve from taking a univariate approach, where individual risk factors are considered in isolation, to more sophisticated analysis of multivariate patterns. At present, there are insufficient high-quality data to allow for this to be done effectively. Further improvement could also come from considering individual risk factors in greater detail. A greater number of risk factors could be considered in terms of more than just a binary presence or absence of risk. More information such as the age at onset of PD in a first degree relative, pack years of smoking or severity of constipation and depression could provide useful information with regards PD risk. New risk associations are also continually being discovered such as dietary preferences, personality traits or history of migraine or epilepsy^11^. Algorithms estimating risk will need to be updated in the presence of information on new risk factors or more robust information on current risk factors, as has recently been the case with the MDS prodromal criteria^3^.

Probability-based algorithms have the potential to offer an effective means of identifying people at highest risk of developing PD with simple, remotely administered tests. This allows for the recruitment of large sample sizes, while identifying individuals who can be targeted for closer monitoring and investigation with more resource-intensive tests. This could be particularly valuable in a research setting, where the low incidence of PD complicates prospective investigation in the years prior to the development of overt, clinically diagnosable PD. The participants providing data in the right tail of the histograms in Fig. 1 are of particular interest for targeting more intensive testing and follow-up. These individuals estimated to be at highest risk could allow identification of pre-diagnostic biomarkers for PD. This is supported by the evidence from the DaT-SPECT data that higher risk estimates are associated with more marked striatal dopamine depletion.

As large prospective cohorts mature, a greater understanding of relationships between pre-diagnostic features with more intensive investigation in high-risk individuals could also provide important information for further refining risk estimation.

## Methods

### Creating an enhanced PREDICT-PD algorithm

Previous PREDICT-PD risk estimation has been based upon single odds ratios, with risk estimates only adjusted if a risk factor was known to be present. To select the most appropriate likelihood ratios to include in an enhanced version of the PREDICT-PD algorithm, we first sought the best available evidence in the published literature. Berg and colleagues^2,3^ have previously published likelihood ratios for a number of PD risk factors which were included in this calculation. However, a number of risk factors included in PREDICT-PD risk estimation had no previous reported positive and negative likelihood ratios for their association with PD. These include head injury and the ever use of any non-steroidal anti-inflammatory medications (NSAIDs), calcium channel blockers (CCB), beta blockers and alcohol. For these, we converted the previously used odds ratios into likelihood ratios using prevalence data from the East London Primary Care database held by the Clinical Effectiveness Group at Queen Mary, University of London (*n* = 1,016,277) (except for alcohol use for which we used data from the UK Office of National Statistics^12^) and Eqs. (2) and (3) (see supplementary material for derivation).

2 $${\rm{LR}} + {\rm{ve}} = \frac{{{\rm{OR}}}}{{((1 - {\rm{prevalence}}) + \left( {{\rm{prevalence}}\;\times\;{\rm{OR}}} \right))}}$$

3 $${\rm{LR}} - {\rm{ve}} = \frac{1}{{(\left( {1 - {\rm{prevalence}}} \right) + \left( {{\rm{prevalence}}\;\times\;{\rm{OR}}} \right))}}$$

After noting that the equation yielding the age-specific risk in the PREDICT-PD algorithm was underestimating risk at older ages (based on a regression of risk against age^1^), we examined whether using the risks based on categories of age in the MDS criteria (which provided more data points to base the regression equation on than previously used and is based on combining data from multiple sources rather than a single source) would lead to a better fit.

We further sought to enhance the PREDICT-PD algorithm’s risk estimation by including continuous, objective motor and non-motor intermediate markers in the risk score. Motor function was determined using the BRAIN tap test^13^. Smell was assessed using the University of Pennsylvania Smell Identification Test (UPSIT)^14^. RBD was assessed subjectively using the REM-sleep behaviour disorder questionnaire (RBDSQ)^15^. In parallel work, likelihood ratios for smell were calculated according to logistic regression models of: (i) the 16 odours identified that were shown to be associated with PD and (ii) a subset of the 6 odours that were most strongly associated with PD. For the BRAIN test, likelihood ratios came from the bivariate Gaussian distributions of delta (difference from the median) kinesia score (KS) and akinesia time (AT) MoM (multiple of the median) values in PD patients and controls. The equations to calculate likelihood ratios based on the 16 odours and the BRAIN test are given in the supplementary material. As part of the same work, 6 questions of the RBDSQ and 7 questions on anxiety and depression were shown to be associated with PD but added little to predicting PD when combined with the 16 or 6 odours associated with PD, likely due to the subjective nature of these tests^8^.

The updated PREDICT-PD risk estimates combine three objective, continuous measures (age, smell, finger tapping) and the remaining factors as subjective, dichotomous measures, and uses likelihood ratios rather than odds ratios. Collectively this new algorithm is referred to as the “enhanced” PREDICT-PD algorithm, whereas scores without these new inclusions and based upon odds ratios are referred to as the basic PREDICT-PD algorithm.

### Data collection

The PREDICT-PD pilot cohort comprised 1,323 healthy 60–80-year old residing in the UK at baseline (mean age 67.2 years, SEM 0.13, 60.9% females) who gave informed consent via the PREDICT-PD website. Exclusion criteria were pre-existing PD, movement disorder, stroke, motor neuron disease, dementia or drug usage known to be associated with iatrogenic Parkinsonism. Participants have completed annual online surveys and a keyboard tapping task up to 6 times over 7 years (416 participants completed the survey all 6 times). For the 5th year of follow-up, no data were collected due to a change in the study website. Detailed information about the recruitment process and data collection methods are described elsewhere^1,4^. If there were missing data in 1 year but available data in subsequent years, we imputed the missing years’ data by assuming risk exposures to be the same as those in the preceding year’s follow-up, but accounting for the fact that they would be one year older (which is associated with a small increase in risk). Similarly smell testing was only done at baseline and in year 3 (892 and 792 completed the UPSIT at baseline and in year 3 respectively), so the results of smell tests at baseline were applied to risk calculations in years 1 and 2, and results of smell tests in year 3 were applied to risk calculations in years 4 and 6. There was little to no missing data for the other risk factors. Pesticide exposure was collected from year 3 onwards; for the preceding years the pesticide exposure was assumed to be the same as in year 3. For those for whom no further follow-up data were available, we did not impute any missing data as we could not be certain of continued consent and that they would return to complete the next survey. Supplementary Table 1 shows the number of participants that had missing survey year data imputed. Incident cases of PD were identified through annual surveys and diagnoses were made through routine clinical care. Newly diagnosed patients were then reviewed in-person by the clinical research team to confirm the diagnosis according to the Queen Square Brain Bank criteria.

### Comparison of risk scores

For each survey year, histograms of risk estimates for each algorithm (basic PREDICT-PD, enhanced PREDICT-PD and MDS criteria) were generated and selected centiles of risk (1st, 2.5th, 10th, 25th, 50th, 75th, 90th, 95th, 97.5th and 99th) were calculated. Risks were expressed as odds and presented graphically on a log scale. We examined the fold difference in risk between the 10th and 90th centile, and the 25th and 75th centile. We did not consider the fold difference in risk between the minimum and maximum, or for example, the 1st and 99th centile as this would be subject to bias from outlying risk estimates or give unstable estimates of fold differences, which is particularly relevant given the inclusion of continuous variables in risk scores (age and the delta KS and AT MoM values). Cox proportional hazard models were used to determine the association between baseline risk scores using each algorithm and incident PD.

### Comparison of risk estimates with DaT-SPECT binding

46 people in the PREDICT-PD pilot cohort had DaT-SPECT imaging. The methods relating to how these images were acquired have been described elsewhere^6,16^. None of the 46 individuals had been diagnosed with PD during continued follow-up. We sought to investigate whether risk estimates were related to striatal dopamine binding. Linear regression was used to investigate the relationship between risk estimates (expressed as log odds) according to each algorithm with each individual’s corresponding striatal DaT-SPECT binding data. Each participant had bilateral DaT-SPECT binding values and we took the lower of the two values as a marker of dopamine depletion. The risk estimates closest in time to the DaT-SPECT imaging were used in the analysis, which were either year 2 or year 3 risks. Confidence intervals for the *R*^2^ value were calculated by bootstrapping, with 5000 replicates.

### Reporting summary

Further information on research design is available in the Nature Research Reporting Summary linked to this article.

## Supplementary information

supplementary material

REPORTING SUMMARY

## Data Availability

Applications for PREDICT-PD data are reviewed by the PREDICT-PD steering committee.

## Code availability

All analyses were performed using Stata version 15 (StataCorp, College Station, Texas). Codes for data analysis in this study are available upon request
