# Supplementary material for: Improving estimation of Parkinson’s disease risk—the enhanced PREDICT-PD algorithm
Source: NPJ Parkinsons Dis. 2021 Apr 1;7:33. doi: 10.1038/s41531-021-00176-9 (PMC8017005; doi:10.1038/s41531-021-00176-9)
Supplement: Supplementary file 1 — supplementary material [file 41531_2021_176_MOESM1_ESM.pdf]

Improving estimation of Parkinson's disease risk - The enhanced PREDICT-PD algorithm: Supplemental material

Derivation of formula to calculate positive and negative likelihood ratios from the odds ratio and prevalence.

Assume we have a standard 2x2 table showing the number of PD and unaffected individuals exposed or not to some factor,

	Exposed	Unexposed
PD	A	B
Unaffected	C	D

then the odds ratio (*OR*) is

$$OR = \frac{A/_B}{C/_D} = \frac{AD}{BC} \qquad (1)$$

and the prevalence (*p*) of the exposure in the unaffected population (which given that PD is not common approximates the prevalence in the population of PD patients and unaffected individuals) is

$$p = \frac{C}{C+D} \qquad (2)$$

The positive likelihood ratio (*LR+*) is the detection rate (proportion of PD patients with the exposure) divided by the false-positive rate (proportion of unaffected individuals with the exposure)

$$LR+= \frac{A/_{(A+B)}}{C/_{(C+D)}} = \frac{A(C+D)}{C(A+B)} = \frac{AD(C+D)}{CD(A+B)} = \frac{\frac{AD}{C(AD+BD)}}{C+D} = \frac{AD}{BC\,\frac{AD+BD}{B(C+D)}}$$

$$= \frac{AD}{BC\left(1-\frac{C}{C+D}+\left(\frac{C}{C+D}\right)\left(\frac{AD}{BC}\right)\right)} = \frac{\frac{AD}{BC}}{\left(1-\frac{C}{C+D}+\left(\frac{C}{C+D}\right)\left(\frac{AD}{BC}\right)\right)}$$

Then from equations 1 and 2

$$= \frac{OR}{(1-p+pOR)}$$

The negative likelihood ratio (*LR-*) is the false negative rate (proportion of PD patients without the exposure) divided by the true-negative rate (proportion of unaffected individuals without the exposure)

$$LR- = \frac{B/(A+B)}{D/(C+D)} = \frac{B(C+D)}{D(A+B)} = \frac{1}{\frac{AD+BD}{B(C+D)}} = \frac{1}{1 - \frac{C}{C+D} + \frac{AD}{B(C+D)}}$$

$$= \frac{1}{\left(1 - \frac{C}{C+D} + \left(\frac{C}{C+D}\right)\left(\frac{AD}{BC}\right)\right)}$$

Then from equations 1 and 2

$$= \frac{1}{(1 - p + pOR)}$$

Formulae to calculate likelihood ratios for smell and the BRAIN test.

The likelihood ratios for the 16-item smell test (LR_{16}) and the 6-item smell test (LR_6) are calculated as follows.[10]

$$LR_{16} = \{8.66404 - (1.492617 \times \text{Gasoline}) - (1.956232 \times \text{Soap}) - (1.436059 \times \text{Watermelon}) - (1.402295 \times \text{Lemon}) - (1.174883 \times \text{Cinnamon}) - (1.084457 \times \text{Natural gas}) - (0.8652221 \times \text{Rose}) - (0.8597572 \times \text{Paint thinner}) - (0.7759867 \times \text{Pineapple}) - (0.6495947 \times \text{Banana}) - (0.5584326 \times \text{Cedar}) - (0.5074133 \times \text{Cherry}) + (0.6116164 \times \text{Strawberry}) - (0.4896007 \times \text{Coconut}) - (0.6320504 \times \text{Menthol}) + (0.5117308 \times \text{Mint})\}/\left(\frac{932}{887}\right)$$

$$LR_6 = \{7.313897 - (1.952729 \times \text{Gasoline}) - (2.232339 \times \text{Soap}) - (1.859984 \times \text{Watermelon}) - (1.511823 \times \text{Lemon}) - (1.544792 \times \text{Cinnamon}) - (1.561454 \times \text{Natural gas})\}/\left(\frac{932}{887}\right)$$

where each odour is equal to one if correctly identified, or 0 if incorrectly identified

BRAIN test kinesia scores and akinesia times are converted to difference from the age and gender-specific median (delta) and multiple of the age and gender-specific median (MoM) values respectively using linear regression (with akinesia times log-transformed). Delta kinesia scores less than -30 are set at -30 and scores above 10 are set at 10. AT MoM values less than 0.747 are set at 0.747 and values above 3.0 are set at 3.0. The likelihood ratio (LR) for delta kinesia score (KS) and $\log_e$ akinesia time MoM (AT) is as follows.[10]

$$LR = \frac{\dfrac{1}{2\pi\sigma_{KS,PD}\sigma_{AT,PD}\sqrt{1-r_{PD}^2}} exp\left(-\dfrac{Z_{PD}}{2(1-r_{PD}^2)}\right)}{\dfrac{1}{2\pi\sigma_{KS,U}\sigma_{AT,U}\sqrt{1-r_U^2}} exp\left(-\dfrac{Z_U}{2(1-r_U^2)}\right)}$$

where

$$Z_{PD} = \frac{\left(KS-\mu_{KS,PD}\right)^2}{\sigma_{KS,PD}^2} - \frac{2r_{PD}\left(KS-\mu_{KS,PD}\right)\left(AT-\mu_{AT,PD}\right)}{\sigma_{KS,PD}\sigma_{AT,PD}} + \frac{\left(AT-\mu_{AT,PD}\right)^2}{\sigma_{AT,PD}^2}$$

and

$$Z_U = \frac{\left(KS-\mu_{KS,U}\right)^2}{\sigma_{KS,U}^2} - \frac{2r_U\left(KS-\mu_{KS,U}\right)\left(AT-\mu_{AT,U}\right)}{\sigma_{KS,U}\sigma_{AT,U}} + \frac{\left(AT-\mu_{AT,U}\right)^2}{\sigma_{AT,U}^2}$$

Where $\mu_{KS,PD}$=-12.80, $\sigma_{KS,PD}$=11.62, $\mu_{AT,PD}$=0.3087, $\sigma_{AT,PD}$=0.3876, r_{PD}=-0.5626, $\mu_{KS,U}$=0, $\sigma_{KS,U}$=9.94, $\mu_{AT,U}$=0, $\sigma_{AT,U}$=0.2701, r_U=-0.3487

Supplementary Figure 1: Risk (expressed as an odds) according to age in the basic PREDICT-PD algorithm (dashed line, open circles) and the revised risk according to age in the enhanced PREDICT-PD algorithm (solid line, filled circles)

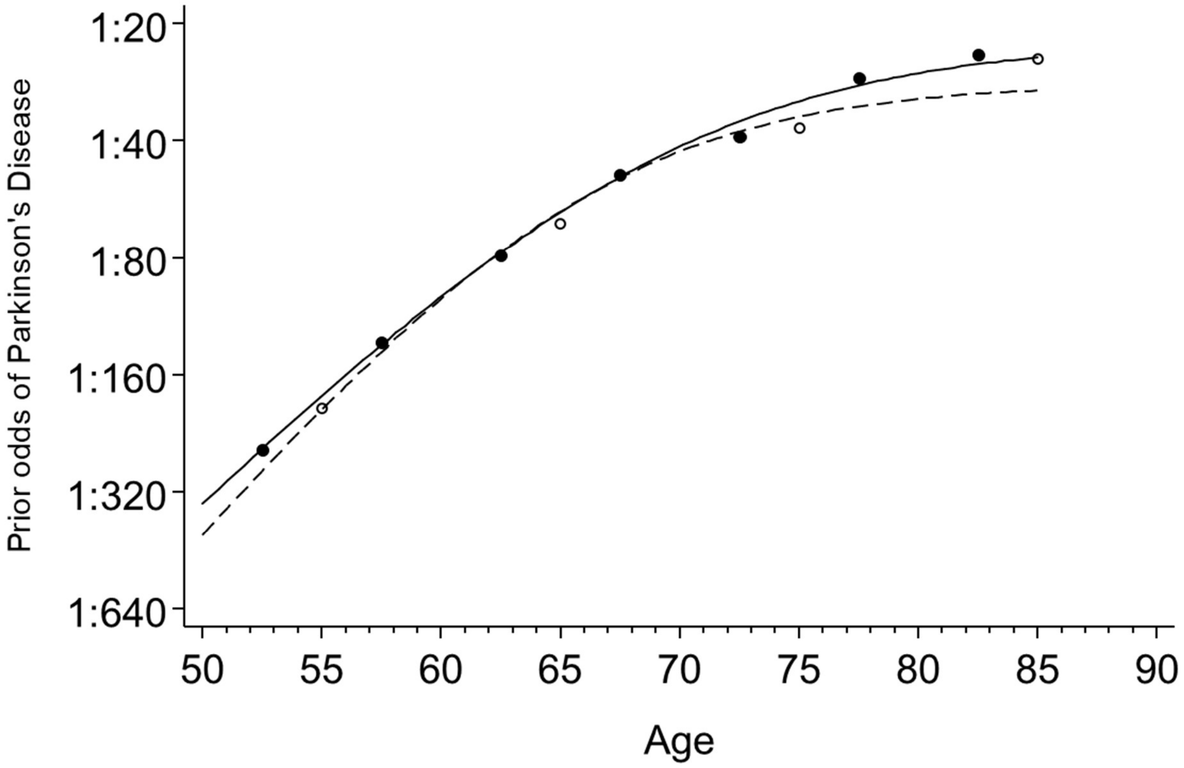

Supplementary Figure 2: Histograms of risk scores for PREDICT-PD participants (presented as odds) at baseline (far left) and in each subsequent survey year with the basic PREDICT-PD algorithm (first row), the enhanced PREDICT-PD algorithm using a 16 item smell test (second row), the enhanced PREDICT-PD algorithm using a 6-item smell test (third row) and the MDS criteria algorithm (fourth row).

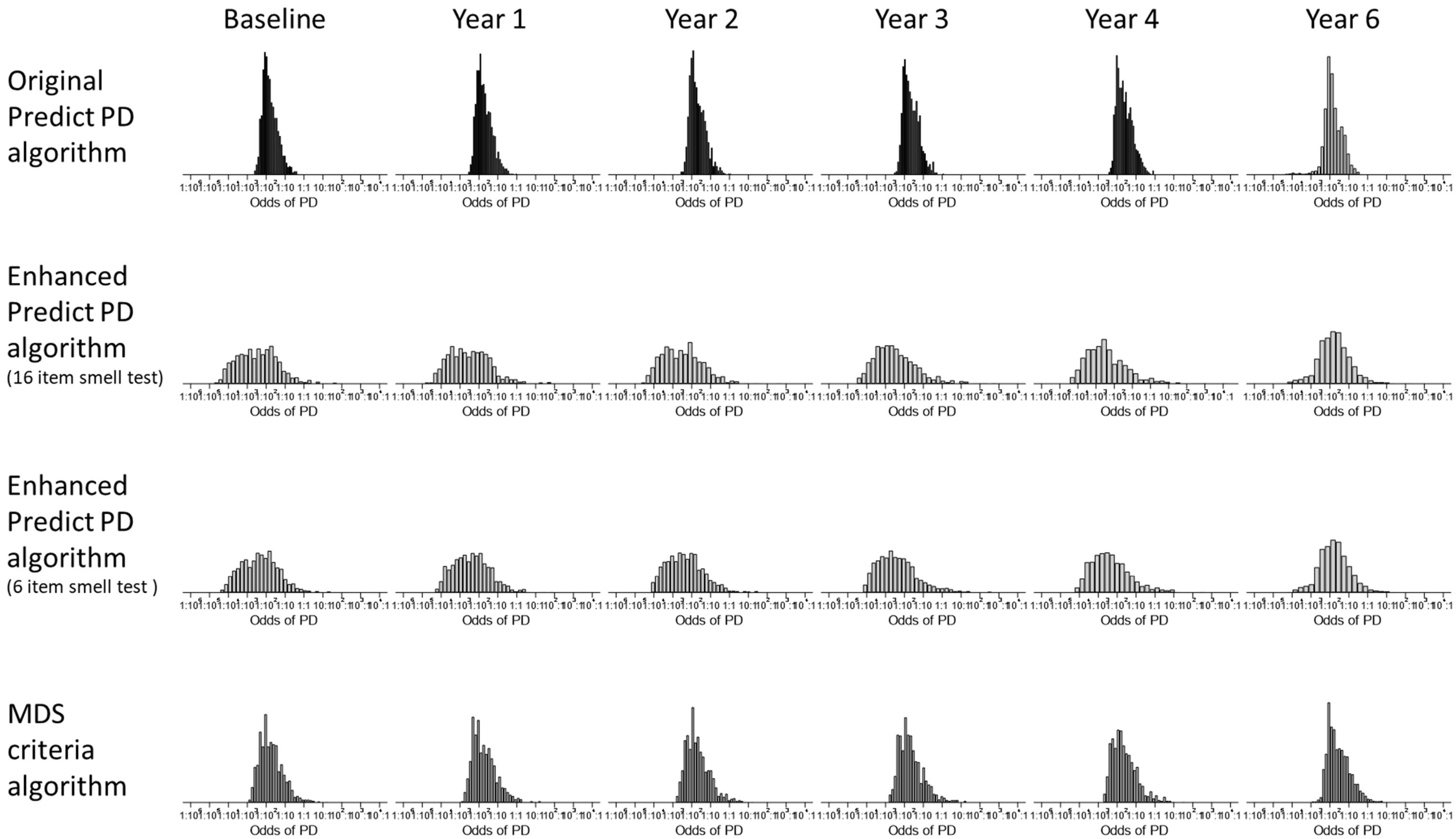

Supplementary Table 1: Full pattern of completed surveys by year: Y=yes survey completed, N=No survey not completed. Grey shaded cells indicate where survey data were imputed using data from the preceding completed year

Baseline	Year 1	Year 2	Year 3	Year 4	Year 6	N
Y	Y	Y	Y	Y	Y	416
Y	Y	Y	Y	Y	N	290
Y	Y	Y	Y	N	Y	13
Y	Y	Y	Y	N	N	44
Y	Y	Y	N	Y	Y	14
Y	Y	Y	N	Y	N	45
Y	Y	Y	N	N	Y	3
Y	Y	Y	N	N	N	69
Y	Y	N	Y	Y	Y	11
Y	Y	N	Y	Y	N	21
Y	Y	N	Y	N	Y	1
Y	Y	N	Y	N	N	10
Y	Y	N	N	Y	Y	4
Y	Y	N	N	Y	N	23
Y	Y	N	N	N	Y	1
Y	Y	N	N	N	N	77
Y	N	Y	Y	Y	Y	10
Y	N	Y	Y	Y	N	13
Y	N	Y	Y	N	Y	1
Y	N	Y	Y	N	N	4
Y	N	Y	N	Y	Y	1
Y	N	Y	N	Y	N	1
Y	N	Y	N	N	Y	1
Y	N	Y	N	N	N	15
Y	N	N	Y	Y	Y	4
Y	N	N	Y	Y	N	6
Y	N	N	Y	N	N	4
Y	N	N	N	Y	Y	2
Y	N	N	N	Y	N	5
Y	N	N	N	N	Y	5
Y	N	N	N	N	N	211

Supplementary Table 2: Selected centiles of the estimated odds of PD according to algorithm and survey year

Survey year	Algorithm	Estimated odds (1:x) of PD at centile of:- 1st	2.5th	10th	25th	50th	75th	90th	97.5th	99th
Baseline	Basic PREDICT-PD	4.1	6.4	17	35	73	122	173	247	317
	Enhanced PREDICT-PD (16-item smell test)	0.4	1.5	14	51	270	1907	6911	17258	24038
	Enhanced PREDICT-PD (6-item smell test)	0.4	1.5	12	46	210	1273	4179	10260	13578
	MDS criteria	0.6	1.8	7.6	24	68	161	288	474	496
Year 1	Basic PREDICT-PD	3.5	5.6	14	29	62	108	154	208	271
	Enhanced PREDICT-PD (16-item smell test)	0.3	1.2	16	56	336	2270	6595	16766	24028
	Enhanced PREDICT-PD (6-item smell test)	0.3	1.5	14	54	246	1270	4155	8894	11952
	MDS criteria	0.6	1.4	6.9	22	71	157	245	376	496
Year 2	Basic PREDICT-PD	3.0	4.8	13	26	59	99	136	190	239
	Enhanced PREDICT-PD (16-item smell test)	0.4	1.6	13	63	362	2086	6335	14977	20647
	Enhanced PREDICT-PD (6-item smell test)	0.4	1.8	11	56	255	1248	3819	8021	11033
	MDS criteria	0.5	1.3	7.0	22	62	142	236	376	440
Year 3	Basic PREDICT-PD	2.8	4.3	12	23	53	93	128	173	218
	Enhanced PREDICT-PD (16-item smell test)	0.1	0.3	9.8	63	421	1921	5712	14789	21845
	Enhanced PREDICT-PD (6-item smell test)	0.1	0.5	8.4	58	308	1446	4101	8215	11574
	MDS criteria	0.3	1.0	5.6	20	60	133	233	376	496
Year 4	Basic PREDICT-PD	2.8	4.3	10	21	48	89	119	159	197
	Enhanced PREDICT-PD (16-item smell test)	0.0	0.3	8.3	57	394	1655	5070	13015	18253
	Enhanced PREDICT-PD (6-item smell test)	0.1	0.3	8.8	56	299	1239	3593	7146	11207
	MDS criteria	0.3	0.8	5.5	18	57	131	233	308	440
Year 6	Basic PREDICT-PD	4.7	6.9	16	34	80	128	207	783	7523
	Enhanced PREDICT-PD (16-item smell test)	0.2	0.9	5.4	19	61	203	503	3812	8234
	Enhanced PREDICT-PD (6-item smell test)	0.2	0.9	5.3	19	61	197	478	2897	6609
	MDS criteria	0.7	1.4	4.9	14	44	100	162	233	292

Supplementary Table 3: Baseline demographics and risk scores among the 10 PREDICT-PD participants that were diagnosed with PD during follow-up

	PD case									
	1	2	3	4	5	6	7	8	9	10
Age	64	67	67	75	70	68	66	65	74	66
Gender	Female	Female	Male	Male	Male	Male	Male	Female	Male	Female
Smoking	Never	Never	Past	Never	Past	Past	Past	Never	Never	Never
Coffee	Yes	Yes	Yes	Yes	Yes	Yes	Yes	Yes	Yes	Yes
Alcohol	No	Yes	Yes	Yes	Yes	Yes	Yes	Yes	Yes	Yes
First degree relative with PD	Yes	Yes	No	No	No	Yes	No	No	No	No
Constipation	No	No	No	No	No	No	No	No	No	No
Erectile dysfunction	N/A	N/A	Yes	No	Yes	Yes	No	N/A	No	N/A
Diabetes	No	No	Yes	No	No	No	No	No	No	No
Head injury	-	No	No	No	No	Yes	No	No	No	No
Beta blocker use	No	No	Yes	No	No	No	No	No	No	No
Calcium channel blocker use	No	No	No	No	No	No	No	No	No	No
NSAID use	No	No	No	No	No	No	No	No	No	No
Moderate to severe depression or anxiety	Yes	No	Yes	No	No	No	No	No	No	No
Pesticide exposure	No	No	No	No	Yes	No	No	No	No	No
BRAIN test										
Kinesia score	46	41	56	-	24	41	57	43	43	49
Akinesia time	101	90	68	-	195	151	99	118	98	136
UPSIT score	10	-	35	-	-	24	29	-	-	-
RBDSQ score	3	0	12	1	0	7	2	2	3	2
HADS score	18	4	7	5	10	2	8	13	5	1
Risk score (1:x)										
Basic PREDICT-PD	25.37	38.62	11.76	57.99	23.55	5.19	114.56	155.06	59.17	135.75
Enhanced PREDICT-PD (16 item smell test)	0.02	23.48	667.72	28.31	0.17	0.03	2339.90	64.91	43.03	79.07
Enhanced PREDICT-PD (6 item smell test)	0.01	23.48	316.04	28.31	0.17	0.04	7462.64	64.91	43.03	79.07
MDS criteria	8.90	6.88	16.31	36.72	2.36	0.14	235.82	27.73	10.49	100.35
